# Proteomic signatures of physical, cognitive, and imaging outcomes in multiple sclerosis

**DOI:** 10.1002/acn3.51996

**Published:** 2024-01-17

**Authors:** Kian Jalaleddini, Dejan Jakimovski, Anisha Keshavan, Shannon McCurdy, Kelly Leyden, Ferhan Qureshi, Atiyeh Ghoreyshi, Niels Bergsland, Michael G. Dwyer, Murali Ramanathan, Bianca Weinstock‐Guttman, Ralph HB Benedict, Robert Zivadinov

**Affiliations:** ^1^ Octave Biosciences Menlo Park California USA; ^2^ Buffalo Neuroimaging Analysis Center, Department of Neurology, Jacobs School of Biomedical Sciences University at Buffalo, State University of New York Buffalo New York USA; ^3^ Department of Pharmaceutical Sciences State University of New York, Buffalo Buffalo New York USA; ^4^ Jacobs MS Center, Department of Neurology, Jacobs School of Biomedical Sciences University at Buffalo, State University of New York Buffalo New York USA; ^5^ Center for Biomedical Imaging at the Clinical Translational Science Institute University at Buffalo, State University of New York Buffalo New York USA

## Abstract

**Background:**

A quantitative measurement of serum proteome biomarkers that would associate with disease progression endpoints can provide risk stratification for persons with multiple sclerosis (PwMS) and supplement the clinical decision‐making process.

**Materials and Methods:**

In total, 202 PwMS were enrolled in a longitudinal study with measurements at two time points with an average follow‐up time of 5.4 years. Clinical measures included the Expanded Disability Status Scale, Timed 25‐foot Walk, 9‐Hole Peg, and Symbol Digit Modalities Tests. Subjects underwent magnetic resonance imaging to determine the volumetric measures of the whole brain, gray matter, deep gray matter, and lateral ventricles. Serum samples were analyzed using a custom immunoassay panel on the Olink™ platform, and concentrations of 18 protein biomarkers were measured. Linear mixed‐effects models and adjustment for multiple comparisons were performed.

**Results:**

Subjects had a significant 55.6% increase in chemokine ligand 20 (9.7 pg/mL vs. 15.1 pg/mL, *p* < 0.001) and neurofilament light polypeptide (10.5 pg/mL vs. 11.5 pg/mL, *p* = 0.003) at the follow‐up time point. Additional changes in CUB domain‐containing protein 1, Contactin 2, Glial fibrillary acidic protein, Myelin oligodendrocyte glycoprotein, and Osteopontin were noted but did not survive multiple comparison correction. Worse clinical performance in the 9‐HPT was associated with neurofilament light polypeptide (*p* = 0.001). Increases in several biomarker candidates were correlated with greater neurodegenerative changes as measured by different brain volumes.

**Conclusion:**

Multiple proteins, selected from a disease activity test that represent diverse biological pathways, are associated with physical, cognitive, and radiographic outcomes. Future studies should determine the utility of multiple protein assays in routine clinical care.

## Introduction

Multiple sclerosis (MS) is a chronic, inflammatory, demyelinating, and degenerative disease of the central nervous system (CNS) resulting in progressive accrual of physical and cognitive disability.^1^ People with multiple sclerosis (pwMS) have heterogeneous clinical presentation and multiple demographic, clinical, and paraclinical risk factors have been associated with poorer long‐term outcomes.^1^ Over the last three decades, a plethora of highly effective disease modifying therapies (DMTs) have been developed that can significantly lessen the long‐term disability.^1^ They generally range from highly effective but immunosuppressive therapies to moderately effective immunomodulators with lower rate of adverse events.^2^ Therefore, development of attainable and cost‐effective biomarkers can provide risk stratification for pwMS and supplement the clinical decision‐making process.

In addition to validated imaging biomarkers such as lesion pathology and whole brain atrophy, recent developments in proteomic assay technology have allowed detection and measurement of picomolar concentrations of blood biomarkers.^3^ These biomarkers can provide proxy measures regarding the occurrence and extent of pathological changes in the CNS.^4^ For example, higher blood levels of neurofilament light chain (NfL), an intermediate filament present in neurons, can indicate greater neuroaxonal destruction, and higher levels of glial fibrillary acidic protein (GFAP) can indicate glial cell activation, both indicative of presence of disease activity.^5^, ^6^ Although these individual biomarkers have already emerged as candidate outcomes measures in MS, a single‐protein biomarker can have limited ability in capturing changes within multiple parallel pathophysiological MS pathways.^7^ When compared to MRI measures, blood‐derived analyses are less costly, more accessible, and can be bundled together with routine clinical blood work.^8^

The multiple sclerosis disease activity (MSDA) is a recently developed and analytically validated^9^ panel of 18 biomarkers that represent changes within four main pathophysiological pathways of neuroinflammation, immunomodulation, myelin biology, and neuroaxonal integrity.^9^ In the first clinical validation study, the MSDA platform was trained and tested as a predictor for presence of gadolinium‐enhancing lesions or new/newly enlarging T2 lesions in a cohort of 614 samples.^10^ The multi‐protein scores outperformed the best individual protein (NfL) with area under curve change from 0.726 to 0.781.^10^ Determining the relation of the MSDA panel to long‐term disability outcomes and examining the longitudinal predictive properties of such an assay are essential for clinical adoption and wide‐spread clinical utility.

The aims of this study were to determine the relationship between the multiple proteomic biomarkers and cross‐sectional and longitudinal MS outcomes, including physical disability, cognitive performance, and conventional MRI outcomes. We hypothesize that more than one proteomic biomarker from multiple pathophysiological pathways would correlate with long‐term clinical and MRI outcomes in a heterogenous group of pwMS.

## Methods

### Study population

A total of 202 patients were assessed in these analyses and derived from a larger longitudinal, study to explore the role of cardiovascular, environmental, and genetic risk factors in multiple sclerosis patients (CEG‐MS).^11^ In particular, for this study, the pwMS were enrolled at Department of Neurology, University at Buffalo, State University of New York at baseline between 2009 and 2012 and returned for a follow‐up visit in years 2014–2017. The inclusion criteria were as follows: (1) baseline age of 18–75 years old; (2) diagnosed with either MS or clinically isolated syndrome (CIS), defined by the 2010‐revised McDonald criteria (which was current at the time of enrollment)^12^; (3) availability of either baseline or follow‐up serum sample, MRI, clinical and neuropsychological assessments within 30 days of each other. The exclusion criteria were as follows: (1) having clinical relapse or receiving intravenous corticosteroid therapy within 30 days before the MRI and serum sampling, (2) not able to undergo any of the aforementioned study procedures, and (3) pregnant or nursing mothers. The CEG‐MS study and the retrospective proteomic analyses were approved by the University at Buffalo Institutional Review Board (IRB), and all subjects provided a signed consent form.

### Physical and cognitive disability measures

A board‐certified neurologist evaluated patients for global disability using the Expanded Disability Status Scale (EDSS) score,^13^ a board‐certified neuropsychologist oversaw a clinical assessment that included assessment of quantitative mobility and leg function, using the Timed 25 Foot Walk Test (T25FWT),^14^ quantitative finger dexterity using the 9‐Hole Peg Test (9HPT),^15^ and cognitive efficiency and speed performance using the Symbol Digit Modalities Test (SDMT) and the Paced Auditory Serial Addition Test (PASAT).^16^ Due to the long follow‐up time, we do not expect significant training effects in the neuropsychological performance. Moreover, alternate test forms were used to minimize the practice effect any further. A structured questionnaire was also used to collect demographic and clinical information. According to the clinical presentation and disease history, pwMS were categorized as CIS, relapsing–remitting MS (RRMS), progressive MS (PMS) (further categorized to primary (PPMS), or secondary progressive (SPMS)).^17^

Presence of disability progression (DP) over the follow‐up was defined using standard criteria of changes in EDSS scores: (1) An increase of 2 or more points if the baseline EDSS was zero; (2) an increase of 1.5 or more points if the baseline EDSS was 0.5, (3) an increase of ≥1 point if the baseline EDSS is between 1.0 and 5.0, and (4) an increase of equal or greater than 0.5 point if the baseline EDSS was ≥5.5.^18^ Worsening in T25FWT and 9HPT was defined as an increase of greater than or equal to 20% from baseline to follow‐up.^14^, ^15^ Worsening in SDMT performance was defined using several previously used criteria: (1) decrease of 4 or more points from baseline to follow‐up^16^; (2) decrease of 8 or more points from baseline to follow‐up; (3) being classified as cognitively impaired based with |*z*‐scores| > 1.5 derived from a healthy control population published in the literature with mean and standard deviation of 55.49, 13.06, respectively.^5^, ^19^ Due to addition of cognitive assessment of the cohort late into the start of the study, a significantly smaller number of subjects received baseline neuropsychological assessment when compared to all subjects at the 5‐year follow‐up visit, Table S1.

### Proteomics analyses

**Figure 1:**
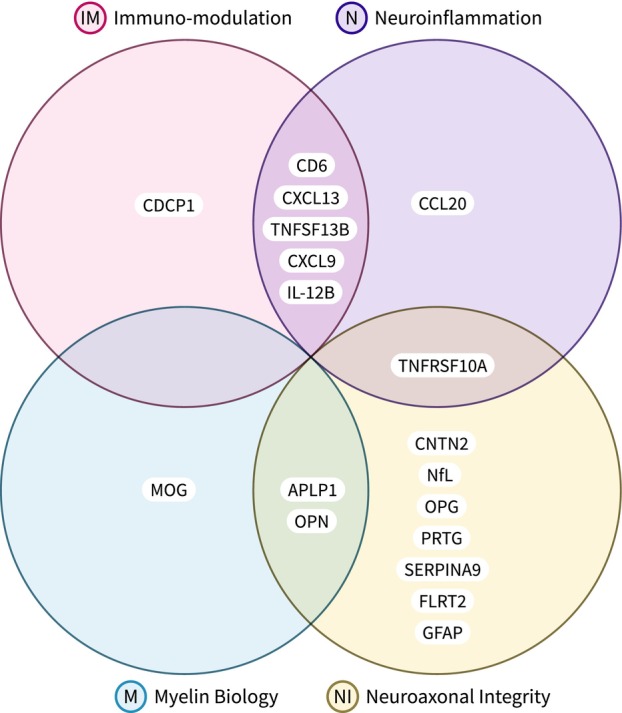
The Octave Bioscience Multiple Sclerosis Disease Activity (MSDA) test was developed using Proximity Extension Assay (PEA) methodology on the Olink™. The custom assay panel measures the concentrations of 21 proteins, and utilizes an algorithm to determine 4 disease pathways scores (immunomodulation, neuroinflammation, myelin biology, and neuroaxonal integrity) and an overall disease activity score. Only 18/21 proteins are used in the MSDA algorithm. GH was excluded due to diurnal variability reasons, COL4A1 due to high intra‐ and inter‐assay coefficients of variation, and VCAN because it did not have strong statistical correlations with endpoints of disease activity and was not determined to be associated with the four biological pathways reported by the MSDA test. In this study, we are using the 18 proteins plus VCAN as we are utilizing the custom assay panel to investigate endpoints beyond disease activity. The full names of the proteins corresponding to the abbreviations are given in Table S8.

During the active study recruitment, the blood samples were processed into serum using serum separator tubes according to manufacturer specifications within 24 h of acquisition by the School of Pharmacy and Pharmaceutical Sciences at University at Buffalo. They were stored at −80°C until further use. After the conclusion of the study, all samples were sent to Octave Bioscience (Menlo Park, CA, USA) for proteomic analysis using the MSDA assay panel and they were analyzed as a single batch.^9^ Inclusion criteria between development studies for MSDA and the CEG cohort were consistent. Proteomic analysis was performed blinded to the demographic, clinical, and MRI data. The MSDA assay uses Proximity Extension Assay (PEA) methodology and is performed on the Olink™ platform. Twenty‐one proteins that are associated with key biological pathways of MS pathophysiology were selected for inclusion on the panel based on results from discovery analyses investigating relative expression of 1196 proteins in previously characterized MS cohorts.^9^ For clarification, we did not utilize the pathway or disease activity scores generated by the MSDA panel in our models. Instead, our analysis concentrated exclusively on the concentration levels of the individual protein biomarkers within the MSDA panel. This distinction is important as our primary focus here is on the progression of the disease, which differs from disease activity and necessitates a distinct analytical approach. The complete list of proteins (with commonly used aliases and their abbreviations) are shown in Fig. 1 and Table S8.

### MRI acquisition and analyses

At baseline and follow‐up visits, pwMS underwent an MRI examination using the same 3 T Signa Excite 12 Twin‐Speed scanner (GE Healthcare, Milwaukee, WI, USA) and eight channel head and neck coil. The standard sequences utilized in these analyses were two‐dimensional (2D) fluid attenuated inversion recovery (FLAIR), 2D T1‐weighted spin echo with and without use of 0.2 mL/kg gadolinium (Gd) contrast acquired 5 min postinjection, and high‐resolution 3D T1‐weighted imaging. The sequence parameters are explained in details elsewhere.^20^

Lesion analysis was performed in a blinded manner with respect to the patient clinical and proteomics status. T2 lesion volume (LV), T1‐LV, and Gd‐LV were obtained using a semi‐automated contouring/thresholding technique using Java Image Manipulation (JIM) version 6.0 (Xinapse Systems Ltd, http://www.xinapse.com/, Essex, UK). The cross‐sectional and longitudinal changes in volumes of brain regions of interest (ROIs) of whole brain (WB), white matter (WM), gray matter (GM), all normalized for head size, were measured using the SIENAX and SIENA algorithms (FMRIB Software Library, http://www.fmrib.ox.ac.uk/fsl).^21^ A lesion inpainting technique was used to avoid tissue misclassification.^22^ Total deep GM (DGM) volume and specific volume of the thalamus were obtained with FMRIB's Integrated Registration and Segmentation Tool (FIRST, https://fsl.fmrib.ox.ac.uk/fsl, version 1.2). The number of patients who had baseline and follow‐up samples are described in Table S1. Lastly, pathological change in whole brain volume was determined as an annualized percent brain volume reduction of greater than or equal to 0.4%^23^ and pathological lateral ventricle volume change if an annualized percent volume expansion of greater than or equal to 3.5%.^24^

The counts presented in Table S1 correspond to patients who have had both baseline and follow‐up blood samples. The table provides demographic information for these patients at different time points and endpoints. Note that all 202 patients in the study cohort who underwent assessments did indeed have baseline blood samples collected.

### Statistical analyses

We employed both Python and R, leveraging Python's versatility for data manipulation and R's specialized statistical packages for rigorous mixed‐effects modeling and post hoc power analysis, ensuring a comprehensive and robust analytical approach. Data and statistical analyses were performed using Python version 3.8.10, SciPy 1.9.3, pandas 1.5.2, pingouin 0.5.3, and NumPy 1.24.1. Ordinary and mixed‐effects models were estimated using R version 4.1.3. Logistic regression models were estimated with Python statsmodels package version 0.13.2. Retrospective power analysis was done using R simr version 1.0.7.

Student's *t*‐test and analysis of covariance (ANCOVA) were used for statistical analysis of parametric continuous variables, and longitudinal analysis was performed using the paired nonparametric Wilcoxon test. Ordinary least‐squares were used to estimate cross‐sectional univariable models; linear mixed‐effects regression models were used to estimate models on longitudinal data; linear logistic regression models were used to estimate dichotomous outcomes (e.g., pwMS disability progression yes/no).

MRI‐based brain volumes, EDSS, and neuropsychological test outcomes were used as dependent variables, and age, sex, BMI, and all proteomic measures as independent predictors (*outcome score = age + sex + body mass index (BMI) + biomarker concentration*) and for the linear mixed‐effects model, subject ID was set as random effect (*outcome score = age + sex + BMI + time point + biomarker concentration + (1|patient ID*). For entry into the regression models, the proteomic data, MRI‐based brain volumes, EDSS, and neuropsychological test scores were transformed using log (10) and all the statistical tests were applied to the log‐transformed data. Logistic regression models were similarly used if the dependent variable was of categorical nature. Outcomes such as R^2^ for ordinary and mixed‐effects regression, McFadden's pseudo‐R^2^ for logistic regression, standardized *β* and *p*‐values were reported. Adjusted *p*‐values lower than 0.05 were considered statistically significant. The regression and correlation *p*‐values underwent false discovery rate (FDR) correction (multiple comparison correction) using the Benjamini–Hochberg procedure. Retrospective power analysis was performed by simulating 1000 new datasets based on the fitted linear mixed‐effects model and assessing the true positives.

Data were visualized using Python matplotlib 3.4.2, seaborn 0.12.1, and plotly 5.12.0 packages. The data distribution was determined using visual inspection of histograms and Q‐Q plots. Volcano plots were used to visualize the significance (*p*‐value) versus effect size.

## Results

### Demographic and clinical characteristics

Table 1 describes the demographics and clinical characteristics (including DMT status, relapse rate, EDSS, MRI metrics) of the pwMS. As expected, the pwPMS were significantly older, had longer disease duration and higher EDSS scores both at baseline and follow‐up visits (*p* < 0.001 for all). We found no statistically significant difference in the rate of disease progression between individuals with clinically isolated syndrome (CIS) or relapsing–remitting multiple sclerosis (RRMS) and those with progressive multiple sclerosis (PMS) (28.6% vs. 37.5%, *p* = 0.251). There were no significant differences in terms of baseline and follow‐up DMT use. As expected, the pwPMS had significantly greater pathology measured by conventional MRI measures of T2‐LV and T1‐LV (*p* < 0.001 and *p* = 0.015) and global measures of WBV, WMV and GMV (*p* < 0.001). The pwCIS/RRMS had on average significantly more Gd lesions when compared to the PMS group (*p* < 0.001). Figure S1 depicts the distribution of disease phenotypes at baseline and transition over the follow‐up. In our study, 73 patients experienced relapses during the follow‐up period. Out of which, 15 patients experienced relapses within 90 days of serum measurement. On average, the time difference between serum measurement and the nearest relapse for this subgroup was 801.51 with a standard deviation of 620.68 days. Among these 15 patients, the average time difference was 51.31 days, with a standard deviation of 27.70 days.

**Table 1:** Demographic, clinical, and conventional MRI characteristics of the study population.

Demographic and clinical characteristics	pwMS (*n* = 202)	CIS/RRMS (*n* = 148)	PMS (*n* = 54)	*p*‐value
Female, *n* (%)	151 (74.8)	106 (71.6)	45 (83.3)	0.09^a^
Age at baseline, mean (SD)	47.1 (11.1)	44.1 (10.6)	55.3 (7.9)	**<0.001** ^b^
Time of follow‐up, mean (SD)	5.4 (0.6)	5.4 (0.6)	5.5 (0.6)	0.732^b^
BMI at baseline, mean (SD)	27.5 (5.8)	27.9 (6.2)	26.5 (4.5)	0.1^b^
Age of disease onset, mean (SD)	32.9 (9.8)	32.6 (9.0)	33.6 (11.8)	0.6^b^
Disease duration at baseline, mean (SD)	13.4 (10.2)	11.1 (8.5)	21.7 (10.5)	**<0.001** ^b^
EDSS at baseline, median (IQR)	2.5 (1.5–5.0)	1.5 (1.5–2.5)	6.0 (4.0–6.5)	**<0.001** ^c^
EDSS at follow‐up, median (IQR)	3.0 (1.6–6.0)	2.0 (1.5–3.5)	6.5 (4.0–6.5)	**<0.001** ^c^
EDSS absolute change, mean (SD)	0.4 (0.9)	0.4 (0.9)	0.4 (0.7)	**<0.001** ^b^
Disability progression, *n* (%)*	56 (30.9)	38 (28.6)	18 (37.5)	0.251^a^
Relapse rate over the follow‐up, mean (SD)	0.172 (0.369	0.204 (0.4)	0.09 (0.24)	**<0.001** ^d^
DMT at baseline, *n* (%)				
IFN‐β	85 (42.1)	60 (40.5)	25 (46.3)	0.271^a^
Glatiramer acetate	37 (18.3)	24 (16.2)	13 (24.1)	
Natalizumab	29 (14.4)	25 (16.9)	4 (7.4)	
Off‐label DMT	5 (2.5)	3 (2.0)	2 (3.7)	
No DMT	46 (22.8)	36 (24.3)	10 (18.5)	
DMT at follow‐up, *n* (%)				
IFN‐β	68 (33.7)	52 (35.1)	16 (29.6)	0.797^a^
Glatiramer acetate	45 (22.3)	31 (20.9)	14 (25.9)	
Natalizumab	15 (7.4)	12 (8.1)	3 (5.6)	
Oral DMT	28 (13.9)	22 (14.9)	6 (11.1)	
Off‐label DMT	12 (5.9)	8 (5.4)	4 (7.4)	
No DMT	34 (16.8)	23 (15.5)	11 (20.4)	
T2‐LV (mL), mean (SD)	13.5 (16.7)	10.3 (14.09)	22.2 (20.1)	**<0.001** ^b^
T1‐LV (mL), mean (SD)	3.0 (7.2)	2.2 (6.44)	5.5 (8.6)	**0.015** ^b^
Gd‐LN, mean (SD)	0.05 (3.2)	0.7 (3.7)	0.04 (0.2)	**<0.001** ^c^
Gd‐LV (mL), mean (SD)	0.07 (0.4)	0.1 (0.48)	0.01 (0.03)	0.194^b^
WBV (mL), mean (SD)	1466.4 (94.3)	1490.2 (87.7)	1401.7 (80.8)	**<0.001** ^b^
WMV (mL), mean (SD)	725.9 (62.2)	738.7 (62.1)	689.8 (46.7)	**<0.001** ^b^
GMV (mL), mean (SD)	740.8 (63.9)	751.4 (65.9)	711.9 (48.1)	**<0.001** ^b^

Thirteen (13) CIS/RRMS patients transitioned into PMS over the follow‐up. Parametric data are shown as mean (standard deviation), whereas nonparametric data are shown as median (interquartile range). The specific comparisons were performed using; a – chi‐square test, b – Student's *t*‐test, c –Mann–Whitney U test, c – Negative binomial regression. *p*‐values lower than 0.05 were considered statistically significant and shown in bold.

BMI, body mass index; CIS, clinically isolated syndrome; DMT, disease modifying therapy; EDSS, Expanded Disability Status Scale; GMV, gray matter volume; IFN, interferon; IQR, interquartile range; LN, lesion number; LV, lesion volume; MS, multiple sclerosis; PMS, progressive multiple sclerosis; RRMS, relapsing–remitting multiple sclerosis; SD, standard deviation; WBV, whole brain volume; WMV, white matter volume.

* Disability progression was available for 181 out of 202 pwMS due to missing EDSS values at either baseline or follow‐up visit.

Out of the pwMS with available longitudinal disability data, 55 out of 181 (30.4%) worsened in EDSS scores, 46 out of 186 (24.7%) had a pathological rate of whole brain atrophy and only 19 out of 186 (10.2%) had a pathological rate of ventricle enlargement, 12 out of 47 (25.5%) worsened in 9HPT, 21 out of 49 (42.8%) worsened in SDMT (4‐points drop), 10 out of 49 (20.4%) worsened in SDMT (8‐points drop), and 9 out of 47 (19.1%) worsened in T25FWT. In total, 42 patients had progression in more than one endpoint metric (excluding SDMT‐8). The paired statistical test revealed that all outcomes worsened significantly at the follow‐up time point except for the WMV and PASAT performance (Table S2). Figure S2 visualizes shifts in MRI‐based volumes, EDSS, and neuropsychological scores between the baseline and follow‐up time points. Figures S3 and S4 demonstrate differences in MRI‐based volumes, EDSS, neuropsychological scores, and biomarker concentrations between pwCIS/RRMS and pwPMS subgroups.

### Proteomic characteristics of the study population

**Figure 2:**
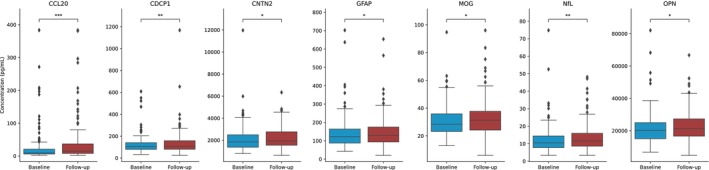
Changes in blood serum biomarker concentration between the baseline and follow‐up time points for those with *p*‐value < 0.05. Paired Wilcoxon signed‐rank test was used to compare between baseline and follow‐up time points. *p*‐value annotation legend: ns: 5.00e‐02 < *p* < = 1.00e+00, *: 1.00e‐02 < *p* < = 5.00e‐02, **: 1.00e‐03 < *p* < = 1.00e‐02, ***: 1.00e‐04 < *p* < = 1.00e‐03, ****: *p* < = 1.00e‐04. Note that only CCL20 and NfL survived the multiple comparison correction.

In total, 202 pwMS had serum samples at the baseline visit and 143 pwMS had serum samples at both the baseline and follow‐up visits. The baseline, follow‐up, and longitudinal change in each of the proteomic biomarkers (shown as median and interquartile range (IQR)) are shown in Fig. 2 for biomarkers with significant shift between the time points and detailed analysis is shown in Table S4. Over the follow‐up, pwMS had a significant 56% increase in CCL20 (9.74 pg/mL vs. 15.1 pg/mL, *p* = 0.001) and 9.4% increase in NfL (10.5 pg/mL vs 11.5 pg/mL, *p* = 0.003). There were also significant shifts in CDCP1, CNTN2, GFAP, MOG, and OPN but they did not survive multiple comparison correction. Figure S4 illustrates a subgroup analysis showing that among the measured proteins, NfL concentration at follow‐up and PRTG concentration at baseline were significantly different in pwPMS compared to pwCIS/RRMS.

In the pwMS who progressed over the follow‐up, there were several proteins whose levels differed between the visits. For the patients who progressed in the 9HPT test, CDCP1 increased by 31% (110 pg/mL vs. 144 pg/mL, *p* = 0.003), TNFRSf10A increased by 37% (5.61 pg/mL vs 7.71 pg/mL, *p* = 0.005), and VCAN increased by 17% (428 pg/mL vs 500 pg/mL, *p* = 0.007). The other proteins in Table 2 did not survive the multiple comparison correction.

**Table 2:** Changes in blood serum protein concentrations between baseline and the follow‐up for pwMS with worsening in disability measures.

Endpoint	Biomarker	Baseline median (IQR)	Follow‐up median (IQR)	Percentage Change (%)	*p*‐value
EDSS worsening	CCL20	11.5 (6.12, 19.3)	15.9 (10.3, 41.5)	38	**0.041**
	CDCP1	108 (81.9, 126)	135 (86.7, 180)	25	**0.005**
	TNFSF13B	4.82 (4.0, 5.95)	5.19 (4.51, 6.27)	7.6	**0.008**
20% 9HPT worsening	CCL20	12.4 (7.68, 19.1)	28.1 (13.9, 42.0)	130	**0.027**
	CDCP1	110 (105, 125)	144 (112, 193)	31	0.003*
	CXCL13	51.7 (37.8, 78.1)	61.9 (51.0, 103)	20	**0.012**
	CXCL9	55.7 (39.1, 77.8)	69.1 (43.9, 101)	24	**0.042**
	OPN	20.9 (16.6, 30.6)	25.2 (19.4, 33.5)	20	**0.042**
	TNFRSF10A	5.61 (4.66, 7.65)	7.71 (6.51, 8.5)	37	0.005*
	VCAN	428 (393, 473)	500 (460, 571)	17	0.007*
4 Points SDMT Worsening	MOG	29.9 (23.0, 38.5)	32.0 (25.2, 43.4)	6.9	**0.027**
8 Points SDMT Worsening	SERPINA9	61.3 (34.3, 77.4)	32.1 (16.1, 59.3)	−48	**0.01**
%0.4 loss in WBV	CDCP1	105 (66.3, 132)	107 (83.7, 147)	1.5	**0.014**
3.5% increase in LVV	SERPINA9	73.0 (57.1, 89.5)	64.4 (38.2, 76.6)	−12	**0.049**

For the list of biomarker, abbreviations refer to Table S8. Wilcoxon signed‐rank tested the significance in shifts. *p*‐values smaller than 0.05 were considered significant and are highlighted with bold fonts, and those with asterisks (*) survived the Benjamini–Hochberg correction for false discovery rate (FDR). All measures are shown as pg/mL except for TNFSF13B and OPN that are shown as ng/mL.

EDSS, Expanded Disability Status Scale; LVV, Lateral Ventricular Volume; SDMT, Symbol Digit Modalities Test; WBV, whole brain volume; 9HPT, 9‐Hole Peg Test.

### Relationship between proteomic data and outcomes in pwMS

#### Longitudinal models

**Figure 3:**
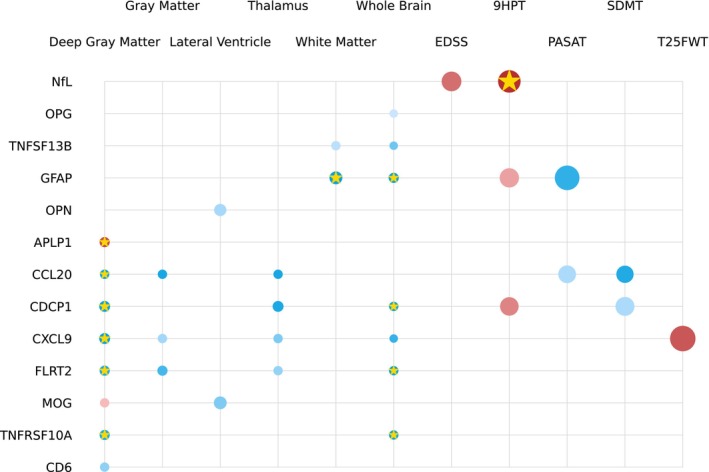
Longitudinal single‐protein model parameters with adjustment for age, sex, and BMI. The radius of each circle is proportional to the estimated standardized coefficient of the corresponding protein; red (blue) circles represent proteins with positive (negative) effects in estimating the second‐class label. The opacity of each circle represents the *p*‐value; a *p*‐value of < 0.001 corresponds to full opacity, and a *p*‐value of 0.05 corresponds to the least opacity. Biomarkers that survived the multiple comparison correction are marked with a gold star (*).

In Fig. 3, we present significant findings from mixed‐effects models that encompassed both time points and all pwMS. Six biomarker candidates – GFAP, FLRT2, CDCP1, TNFRSF10A, CXCL9, and CCL20 – showed associations with changes in brain volumes. Notably, GFAP (*p* = 0.003), FLRT2 (*p* = 0.001), CDCP1 (*p* = 0.004), and TNFRSF10A (*p* = 0.003) were linked to reductions in whole brain volume (WBV) and withstood multiple comparison correction. In the case of white matter volume (WMV), GFAP emerged as the sole significant protein (*p* < 0.001). Additionally, we observed significant associations with DGM volume, including CCL20 (*p* = 0.004), CXCL9 (*p* = 0.001), CDCP1 (*p* = 0.002), FLRT2 (*p* = 0.008), and TNFRSF10A (*p* = 0.009). In terms of clinical and cognitive assessments, worsening in the 9‐HPT score was linked to increased NfL (*p* = 0.001), and these associations held after multiple comparison correction. Detailed results, including estimated coefficients, *p*‐values, and the quality of fit, are provided in Table 3, which exclusively includes significant biomarkers (*p* < 0.05).

**Table 3:** Parameters of the longitudinal linear mixed‐effects model predicting outcome score using single‐protein models consisting of biomarker protein concentration, age, sex, BMI, and time point that passed the significant threshold of *p* < 0.05.

Endpoint	Biomarker	Estimate	R‐squared	*p*‐value	Observed statistical power (95% confidence interval)
DGMV	CD6	−0.003	0.15	0.035	(0.524–0.586)
	TNFRSF10A	−0.004	0.17	0.009*	(0.74–0.793)
	MOG	0.003	0.18	0.045	(0.493–0.555)
	FLRT2	−0.004	0.19	0.008*	(0.743–0.796)
	CXCL9	−0.005	0.16	0.001*	(0.891–0.927)
	CDCP1	−0.005	0.18	0.002*	(0.864–0.904)
	CCL20	−0.003	0.16	0.004*	(0.802–0.85)
	APLP1	0.004	0.17	0.011*	(0.684–0.741)
GMV	FLRT2	−0.004	0.27	0.017	(0.629–0.688)
	CXCL9	−0.003	0.25	0.04	(0.498–0.56)
	CCL20	−0.003	0.26	0.006	(0.748–0.801)
LVV	MOG	−0.008	0.14	0.031	(0.548–0.61)
	OPN	−0.007	0.14	0.041	(0.515–0.577)
Thalamus	CCL20	−0.003	0.16	0.006	(0.747–0.8)
	CXCL9	−0.003	0.15	0.031	(0.553–0.615)
	FLRT2	−0.003	0.18	0.037	(0.499–0.561)
	CDCP1	−0.005	0.17	0.007	(0.754–0.806)
WMV	GFAP	−0.008	0.12	<0.001*	(0.962–0.983)
	TNFSF13B	−0.003	0.11	0.046	(0.5–0.562)
WBV	CDCP1	−0.003	0.24	0.004*	(0.791–0.84)
	CXCL9	−0.002	0.23	0.014	(0.666–0.724)
	FLRT2	−0.003	0.25	0.001*	(0.916–0.948)
	GFAP	−0.004	0.28	<0.001*	(0.956–0.979)
	OPG	−0.002	0.23	0.05	(0.492–0.554)
	TNFSF13B	−0.002	0.24	0.028	(0.559–0.621)
	TNFRSF10A	−0.003	0.24	0.003*	(0.801–0.849)
EDSS	NfL	0.023	0.24	0.021	(0.607–0.668)
9HPT	NfL	0.031	0.21	0.001*	(0.886–0.923)
	GFAP	0.022	0.2	0.037	(0.525–0.587)
	CDCP1	0.02	0.16	0.028	(0.558–0.62)
PASAT	GFAP	−0.038	0.07	0.012	(0.704–0.76)
	CCL20	−0.018	0.02	0.042	(0.488–0.55)
SDMT	CDCP1	−0.021	0.11	0.042	(0.498–0.56)
	CCL20	−0.017	0.09	0.008	(0.758–0.81)
T25FWT	CXCL9	0.041	0.08	0.014	(0.691–0.748)

Refer to Table S8 for the list of protein abbreviations. *p*‐values smaller than 0.05 were considered significant and those with asterisks (*) survived the Benjamini–Hochberg correction for false discovery rate (FDR).

DGMV, deep gray matter volume; EDSS, Expanded Disability Status Scale; GMV, gray matter volume; LVV, lateral ventricular volume; PASAT, Paced Auditory Serial Addition Test; SDMT, Symbol Digit Modalities Test; T25FWT, Timed 25‐Foot Walk Test; WMV, white matter volume; WBV, whole brain volume; 9HPT, 9‐Hole Peg Test.

#### Cross‐sectional models

**Figure 4:**
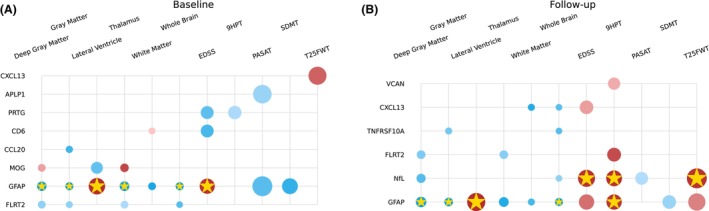
Cross‐sectional single‐protein model parameters with adjustment for age, sex, and BMI for baseline (left) and follow‐up (right). The radius of each circle is proportional to the estimated standardized coefficient of the corresponding protein; red (blue) circles represent proteins with positive (negative) effects in estimating the second‐class label. The opacity of each circle represents the *p*‐value; a *p*‐value < 0.001 corresponds to full opacity, and a *p*‐value of 0.05 corresponds to the least opacity. Biomarkers that survived the multiple comparison correction are marked with a gold star (*).

Figure 4, Tables 4 and S5 present significant findings from linear single‐protein models evaluated individually for baseline and follow‐up outcomes. At baseline, elevated GFAP levels correlated with lower WBV (*p* < 0.001), GMV (*p* < 0.001), thalamic volume (*p* < 0.001), and DGMV (*p* < 0.001), and higher LVV (*p* < 0.001). Furthermore, higher GFAP levels were associated with higher EDSS scores (*p* = 0.002).

**Table 4:** Parameters of the cross‐sectional linear model predicting *baseline* outcome score using single‐protein models consisting of *baseline* biomarker protein concentration, age, sex, and BMI that passed the *p* = 0.05 significance threshold.

Endpoint	Biomarker	Estimate	R‐squared	*p*‐value
DGMV	FLRT2	−0.008	0.16	0.035
	GFAP	−0.015	0.19	<0.001*
	MOG	0.008	0.15	0.037
GMV	CCL20	−0.006	0.25	0.016
	GFAP	−0.009	0.33	<0.001*
	FLRT2	−0.005	0.25	0.036
LVV	MOG	−0.028	0.19	0.024
	GFAP	0.060	0.26	<0.001*
Thalamus	MOG	0.011	0.17	0.008
	GFAP	−0.016	0.18	0.001*
	FLRT2	−0.008	0.16	0.043
WMV	GFAP	−0.008	0.1	0.006
	CD6	0.005	0.1	0.048
WBV	GFAP	−0.009	0.29	<0.001*
	FLRT2	−0.004	0.25	0.021
EDSS	PRTG	−0.034	0.23	0.023
	GFAP	0.050	0.24	0.002*
	CD6	−0.034	0.23	0.018
9HPT	PRTG	−0.037	0.25	0.043
PASAT	GFAP	−0.092	0.11	0.022
	APLP1	−0.079	0.09	0.038
SDMT	GFAP	−0.055	0.18	0.013
T25FWT	CXCL13	0.074	0.17	0.017

Refer to Table S8 for the list of protein abbreviations. *p*‐values smaller than 0.05 were considered significant and those with asterisks (*) survived the Benjamini–Hochberg correction for false discovery rate (FDR).

DGMV, deep gray matter volume; EDSS, Expanded Disability Status Scale; GMV, gray matter volume; LVV, lateral ventricular volume; PASAT, Paced Auditory Serial Addition Test; SDMT, Symbol Digit Modalities Test; T25FWT, Timed 25‐Foot Walk Test; WMV, white matter volume; WBV, whole brain volume; 9HPT, 9‐Hole Peg Test.

At follow‐up, GFAP levels remained correlated with lower WBV (*p* = 0.001), GMV (*p* = 0.001), and DGMV (*p* = 0.002), as well as higher LVV (*p* < 0.001). Both NfL (*p* = 0.001) and GFAP (*p* = 0.003) were linked to worse 9HPT scores, and NfL also correlated with T25FWT (*p* = 0.002) and EDSS scores (*p* = 0.002).

### The association between shifts in biomarker levels and clinical change

**Figure 5:**
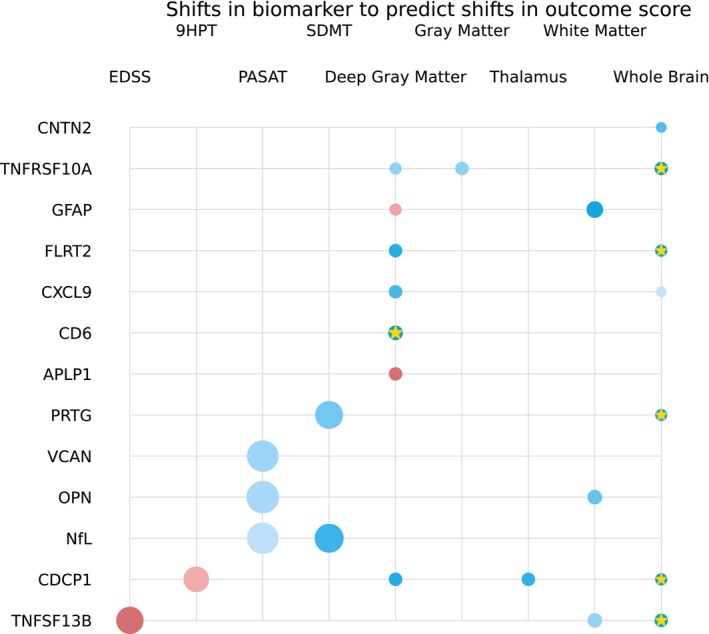
Single‐protein model parameters predicting shifts in outcome score using shifts in biomarker concentration with adjustment for age, sex, and BMI. The radius of each circle is proportional to the estimated standardized coefficient of the corresponding protein; red (blue) circles represent proteins with positive (negative) effects in estimating the second‐class label. The opacity of each circle represents the *p*‐value; a *p*‐value < 0.001 corresponds to full opacity, and a *p*‐value of 0.05 corresponds to the least opacity. Biomarkers that survived the multiple comparison correction are marked with a gold star (*).

To assess the potential impact of shifts in biomarker concentrations on clinical outcomes, we employed a model with shifts in biomarkers between baseline and follow‐up, alongside age, sex, and BMI as independent predictors, and shifts in outcome scores as dependent variables. Notable findings include CDCP1 (*p* = 0.001), FLRT2 (*p* = 0.001), PRTG (*p* = 0.008), TNFRSF10A (*p* < 0.001), and TNFSF13B (*p* < 0.001) as predictors of shifts in WBV. Additionally, CD6 (*p* = 0.001) was identified as a predictor of shifts in DGMV. Further details of these analyses are presented in Fig. 5 and Table S6.

### Baseline biomarker levels as predictors of follow‐up clinical endpoints

**Figure 6:**
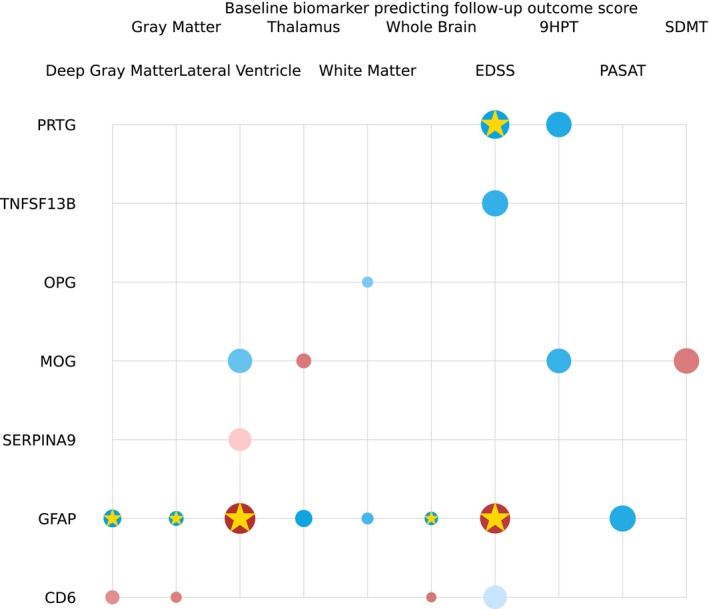
Single‐protein model parameters predicting clinical outcome score at the follow‐up time point using biomarker concentration at the baseline time point with adjustment for age, sex, and BMI. The radius of each circle is proportional to the estimated standardized coefficient of the corresponding protein; red (blue) circles represent proteins with positive (negative) effects in estimating the second‐class label. The opacity of each circle represents the *p*‐value; a *p*‐value < 0.001 corresponds to full opacity, and a *p*‐value of 0.05 corresponds to the least opacity. Biomarkers that survived the multiple comparison correction are marked with a gold star (*).

We assessed the predictive potential of baseline proteomics results for subsequent outcomes and disease progression with significant predictors shown in Fig. 6. While a multitude of proteins initially displayed significance with *p*‐values below 0.05, it is noteworthy that two proteins, GFAP and PRTG, emerged as particularly robust predictors after multiple comparison corrections were applied. As shown in Fig. 6 and Table S7, these proteins consistently exhibited the ability to forecast important clinical and imaging endpoints, including EDSS scores and MRI metrics.

## Discussion

The findings of this longitudinal proteomics study are multifold. Firstly, multiple proteomic biomarkers representing different pathophysiological MS pathways are differentially associated with phenotypical and macroscopic pathological changes. Secondly, worse physical and cognitive outcomes in pwMS were associated with blood‐based measures of NfL. Thirdly, baseline levels of GFAP and PRTG are significant predictors of development of future disability progression (as measured by increase in EDSS scores) and greater neurodegeneration as measured by lateral ventricular expansion. Lastly, worse neurodegenerative MRI outcomes were associated with a greater number of biomarkers including GFAP, CDCP1, CXCL9, CCL20, APLP1, FLRT2, PRTG, аnd TNFRSF10A. The heterogeneous sample of pwMS utilized in this study aimed at closely mirroring the wide heterogeneity seen in the real‐world settings.

The NfL/GFAP relationship with clinical outcomes was recently demonstrated in a similar longitudinal Swiss study.^25^ Over an average follow‐up of 7 years, serum GFAP levels were prognostic of progression independent of relapse activity (PIRA) and complementary to the serum NfL data.^25^ Moreover, NfL levels were prognostic of atrophy in the WMV, whereas GFAP specifically prognosticate GM atrophy.^25^ The multi‐protein panel employed in our study was also utilized in a study of 431 unique pwMS and successfully predicted the real‐world disability status (patient‐reported disability score and patient‐reported outcomes).^26^ The proteomic profiles consistently outperformed individual top‐ranking markers such as NfL and GFAP.^26^ The fact that the same protein biomarkers implicated in their stacking classification algorithm (CDCP1, IL‐12B, and PRTG) were also seen in our objective disability findings further validates the utility and need of multi‐protein proteomic assay.^26^

The stacking of multiple proteomic biomarkers as indicators of different pathophysiological pathways (immunomodulation, neuroinflammation, myelin biology, and neuroaxonal integrity) also provides the potential to stratify pwMS in pathology‐based phenotypes. These proteomic phenotypes may potentially differentiate pwMS that are experiencing disease progression that is driven by neuroinflammatory or neurodegenerative processes and allow more specific treatment allocation. As an area of future work, such patient stratification would be an additional step toward personalized medicine and improved treatment decision‐making process throughout the entire MS disease duration. Albeit nonspecific, the assay does provide simultaneous information for both CNS‐based pathology (MOG, sNfL, and GFAP) and peripheral immune activation (CCL20, CXCL9/13, TNFSF13B, and IL‐12B). Therefore, the multidimensional proteomic information can also be utilized as a potential treatment response marker and indicator of disease control.

We further expand on the literature by demonstrating that the similar set of biomarkers (APLP1, CDCP1, FLRT2, TNFRSF10A, and CCL20) are also relevant to MRI‐based volumetric measures. Of note, the directionality of APLP1‐DGMV relationship (decrease in the biomarker concentration was associated with decreased DGMV) was opposite when compared to the remaining ones. Currently, there are no comprehensive proteomic studies that investigate associations with MRI measures in pwMS. The literature most commonly describes individual associations with one or two proteomic measures (NfL and GFAP).^20^, ^27^, ^28^ Despite the high collinearity between serum NfL and GFAP levels, a cross‐sectional study of 129 pwMS showed that the amount of lesion pathology (T2‐LV) and WM/GM volumes were associated only with GFAP levels and not with NfL.^29^ We corroborate these findings with GFAP remaining a strong predictor all MRI measures acquired in our study (WBV, DGM, LVV, and thalamic volume). Moreover, early measurement of GFAP may be utilized as a significant predictor of future neurodegenerative development, as demonstrated in our study where baseline GFAP was indicative of future LVV expansion. Serum GFAP levels were also recently associated with greater microstructural pathology in 62 pwMS assessed by diffusion tensor imaging.^30^ Our results showed that CCL20 has strong association with the DGM volume and it was increased at the follow‐up time point. This protein was previously shown to be increased in PwMS and specifically with a progression index and was higher during remission than in relapse periods.^31^, ^32^ The retrospective power analysis revealed that the power was sufficient for the majority of the biomarkers that survived the multiple comparison correction. However, the tests were not adequately powered for biomarkers that did not survive the multiple comparison correction (Table 3). This suggests that the sample size and/or effect size could be too small that can have implications for the replicability of these biomarkers in other studies.

The multiplex assays could broaden our understanding of key mechanisms underlying progression by taking a biological‐based approach to objectively quantify disease progression.^33^ They have been demonstrated in other neurological disorders as well.^34^, ^35^ For example, proteomic data from only 4‐Plex assay (NfL, GFAP, tau protein, and ubiquitin c‐terminal hydrolase L1; UCH‐L1) better classified people with traumatic brain injury when compared to only single proteomic measure.^34^ Similarly, cognitive performance in Alzheimer's disease and amyloid PET status can be predicted and classified by a combination of GFAP, amyloid beta, and neurofilament light chain.^35^

While cutoffs of normal versus pathological levels of NfL in pwMS have been previously published,^36^, ^37^ this information is not available for the majority of proteomic biomarkers utilized in this multi‐protein assay. A limited number of studies report the reference intervals and preanalytical GFAP levels.^38^, ^39^ For example, a Danish‐based analysis of 371 apparently healthy subjects reported fairly large ranges with GFAP levels of 25–136 ng/L (20–39 years old), 34–242 ng/L (40–64 years old), and 4–438 ng/L (for 65–90 years old).^38^ Moreover, there was ~10% variability after three freeze–thaw cycles or storing serum samples at −20 °C for an average of 133 days.^38^ Significant semidiurnal variations in GFAP have been reported (9 AM vs. 12 PM vs 9 PM blood draw).^40^ Based on these references, none of the median pwMS values would be considered “pathological.” Other biomarkers such as contactin‐1 may be more susceptible to preanalytical factors and have even greater variability.^41^ After the selection of best performing biomarkers and creation of multi‐protein scores, future studies should aim at determining appropriate cutoffs for best differentiation between normal and pathological states.

In this study, we employed ordinary and linear mixed‐models (LMM) as the primary statistical approaches to explore the relationships between serum biomarker concentrations and various disease progression metrics in MS. While these models offer valuable insights into these associations, we acknowledge that the underlying interactions between biomarkers and disease progression may exhibit nonlinear or more complex patterns. An area of future work is to consider regularized and/or nonlinear regression techniques to capture more complex interactions of serum biomarker concentrations and metrics of disease progression. In addition, multi‐protein regression approaches in cross‐validation studies can be considered to integrate the predictive power of individual biomarkers in one model.^42^ The shifts in DP outcome measures were not uniform. Such imbalance in the training data can deteriorate generalizability of the model.^43^, ^44^ In the future, it will be of interest to balance the data using up, down sampling techniques or using weighted learners. The significantly lower number of available cognitive measures (only 25% of the pwMS) for the baseline time point presents as another study limitation. Moreover, the initial determination of biomarkers within the assay were based on ability to predict presence of contrast‐enhancing and new/newly enlarging lesions.^9^ In comparison with younger more active pwMS from the literature, our population was relatively older and had very limited neuroinflammatory activity. Future development of a more comprehensive assay that contains proteins specific to neurodegenerative changes (vs. neuroinflammation) could better predict the occurrence of long‐term disability worsening. Moreover, the use and change in DMT should be incorporated in future statistical analyses. An additional limitation of our analysis is the lack of a third clinical visit that would allow confirmation of the disease progression and lack of short‐term serial blood samples that would allow better determination of the temporal changes in both the proteomic biomarkers and their relationship with clinical/MRI outcomes. This limitation is particular important when interpreting the relationships in the concurrent proteomic and clinical changes as shown in our manuscript. The proteomic changes that occur are at a significantly smaller timescale when compared to the rate of disability progression or occurrence of significant neurodegenerative changes that can be captured by the current MRI technology. Lastly, the lack of healthy control data does not allow us to determine pathological protein cutoffs and risk stratify the pwMS based on healthy condition.

As part of our future research, we acknowledge the potential to extend our current findings by developing predictive models that utilize machine learning approaches. Specifically, we plan to explore the construction of predictive models that can harness the information contained in baseline biomarker levels to forecast disease progression in MS patients. This entails the use of comprehensive machine learning techniques, including feature selection, model training, and validation, to build robust predictive models. Moreover, the individual significant findings in our analysis should only be interpreted as one component of a repeating pattern of findings rather than focusing on specific biomarker‐outcome correlation. The proteomic levels in the serum can be highly variable and influenced by many biological factors.

In conclusion, the clinical, cognitive, and MRI‐based outcomes in pwMS are associated with more than one proteomic biomarker. While sNfL had the strongest associations with physical disability such as EDSS scores and hand dexterity, additional proteomic biomarkers related to neuroaxonal integrity were associated with cross‐sectional and longitudinal MRI measures of brain atrophy. Multi‐protein assays may be essential in capturing the complex MS pathophysiology as part of the disease stratification, monitoring and potentially utilized as predictors of future accrual of pathology. Before implementation into routine clinical practice, future studies should determine the treatment responsiveness of such proteomic biomarkers. Moreover, creation of a composite score out of proteomic biomarkers that have been proven as good predictors could further ease their clinical implementation.

## Author Contributions

*Conceptualization*, Kian Jalaleddini, Ferhan Qureshi, Dejan Jakimovski and Robert Zivadinov; *Methodology*, Kian Jalaleddini, Dejan Jakimovski, Anisha Keshavan, Shannon McCurdy, Kelly Leyden, Ferhan Qureshi, Atiyeh Ghoreyshi, Niels Bergsland, Michael G. Dwyer, Murali Ramanathan, Bianca Weinstock‐Guttman, Ralph H. B. Benedict, Robert Zivadinov; *Software*, Michael G. Dwyer and Niels Bergsland; *Formal analysis*, Kian Jalaleddini; *Investigation*, Kian Jalaleddini, Dejan Jakimovski, Anisha Keshavan, Shannon McCurdy, Kelly Leyden, Ferhan Qureshi, Atiyeh Ghoreyshi, Niels Bergsland, Michael G. Dwyer, Murali Ramanathan, Bianca Weinstock‐Guttman, Ralph H. B. Benedict, Robert Zivadinov; *Resources*, Robert Zivadinov; *Data curation*, Kian Jalaleddini, Dejan Jakimovski, Anisha Keshavan, Shannon McCurdy, Kelly Leyden, Ferhan Qureshi, Atiyeh Ghoreyshi, Niels Bergsland, Michael G. Dwyer, Murali Ramanathan, Bianca Weinstock‐Guttman, Ralph H. B. Benedict, Robert Zivadinov; *Writing – original draft preparation*, Kian Jalaleddini, Dejan Jakimovski; *Writing – review and editing*: Kian Jalaleddini, Dejan Jakimovski, Anisha Keshavan, Shannon McCurdy, Kelly Leyden, Ferhan Qureshi, Atiyeh Ghoreyshi, Niels Bergsland, Michael G. Dwyer, Murali Ramanathan, Bianca Weinstock‐Guttman, Ralph HB Benedict, Robert Zivadinov; *Supervision*, Kian Jalaleddini, Dejan Jakimovski, Ferhan Qureshi and Robert Zivadinov; *Project administration*, Kian Jalaleddini, Dejan Jakimovski, Anisha Keshavan, Shannon McCurdy, Kelly Leyden, Ferhan Qureshi, Atiyeh Ghoreyshi, Niels Bergsland, Michael G. Dwyer, Murali Ramanathan, Bianca Weinstock‐Guttman, Ralph H. B. Benedict, Robert Zivadinov; *Funding acquisition*, Robert Zivadinov. All authors have read and agreed to the published version of the manuscript.

## Conflict of Interest Statement

Kian Jalaleddini, Ferhan Qureshi, Anisha Keshavan Shannon McCurdy, Kelly Leyden, and Ati Ghoreyshi are employees of and either hold stock or stock options at Octave Bioscience. Dejan Jakimovski received honoraria for serving on the advisory board of AstraZeneca. He also serves as an Associate Editor for Clinical Neurology and Neurosurgery and compensated by Elsevier B.V. Niels Bergsland has nothing to disclose. Murali Ramanathan received research funding from the National Multiple Sclerosis Society, Department of Defense and National Institute of Neurological Diseases and Stroke. Michael G. Dwyer received compensation from Keystone Heart for consultant fees. He received financial support for research activities from Bristol Myers Squibb, Mapi Pharma, Keystone Heart, Protembis, and V‐WAVE Medical. Bianca Weinstock‐Guttman received honoraria for serving in advisory boards and educational programs from Biogen Idec, Novartis, Genentech, Genzyme and Sanofi, Janssen, Abbvie, and Bayer. She also received support for research activities from the National Institutes of Health, National Multiple Sclerosis Society, Department of Defense, and Biogen Idec, Novartis, Genentech, Genzyme, and Sanofi. Ralph HB Benedict received honoraria, speaking, or consulting fees from BMS, EMD Serono, Genentech, Novartis, Roche, and Sanofi, and has received research support from BMS, National Institutes of Health and National Multiple Sclerosis Society. He has received royalties from Psychological Assessment resources, Inc. Robert Zivadinov has received personal compensation from Bristol Myers Squibb, EMD Serono, Sanofi, Keystone Heart, Protembis, and Novartis for speaking and consultant fees. He received financial support for research activities from Sanofi, Novartis, Bristol Myers Squibb, Octave, Mapi Pharma, Keystone Heart, Protembis, and V‐WAVE Medical.

## Supporting information


Supplementary Figure 1.

Supplementary Table 1.
