# Supplementary material for: Proteomic signatures of physical, cognitive, and imaging outcomes in multiple sclerosis
Source: Ann Clin Transl Neurol. 2024 Jan 17;11(3):729–43. doi: 10.1002/acn3.51996 (PMC10963282; doi:10.1002/acn3.51996)
Supplement: Supplementary file 1 — Supplementary Figure 1. [file ACN3-11-729-s001.docx]

**Supplement Figure 1.** Patients might change disease course from baseline (left) to follow-up (right) time points.


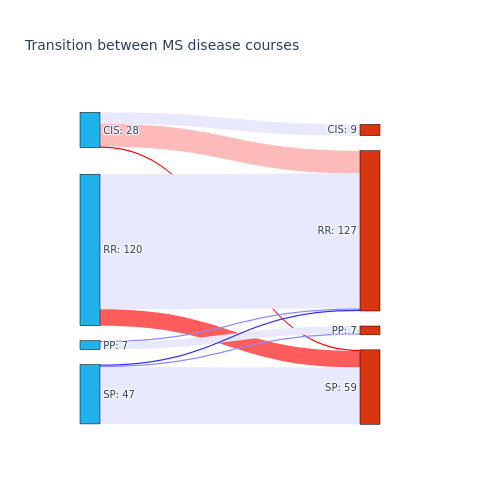


**Legend:** RR - relapsing-remitting multiple sclerosis, CIS - clinically isolated syndrome, SPMS - secondary progressive multiple sclerosis, PPMS - primary progressive multiple sclerosis.

**Supplement Figure 2.** Changes in brain MRI volumes, Expanded Disability Status Scale (EDSS), and neuropsychological scores between baseline and over the follow-up.


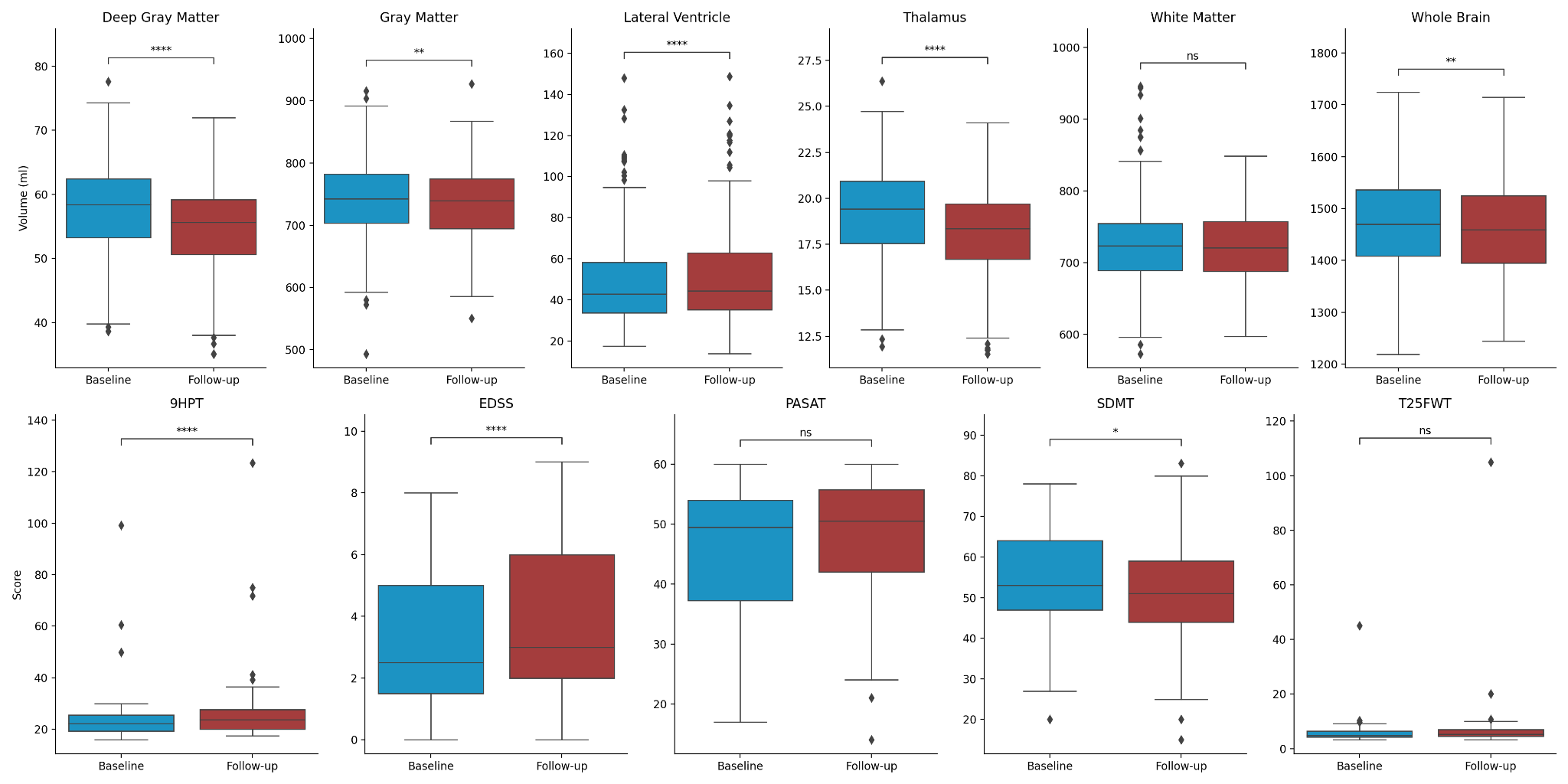


**Legend:** Paired Wilcoxon signed-rank test was used to compare between baseline and follow-up time points. p-value annotation legend: ns: 5.00e-02 < p <= 1.00e+00, *: 1.00e-02 < p <= 5.00e-02, **: 1.00e-03 < p <= 1.00e-02, ***: 1.00e-04 < p <= 1.00e-03, ****: p <= 1.00e-04.

**Supplement Figure 3.** Changes in brain MRI volume, Expanded Disability Status Scale (EDSS), and neuropsychological scores between patients with Progressive MS (PMS) and Clinically Isolated Syndrome (CIS) and Relapsing-Remitting MS(RRMS).


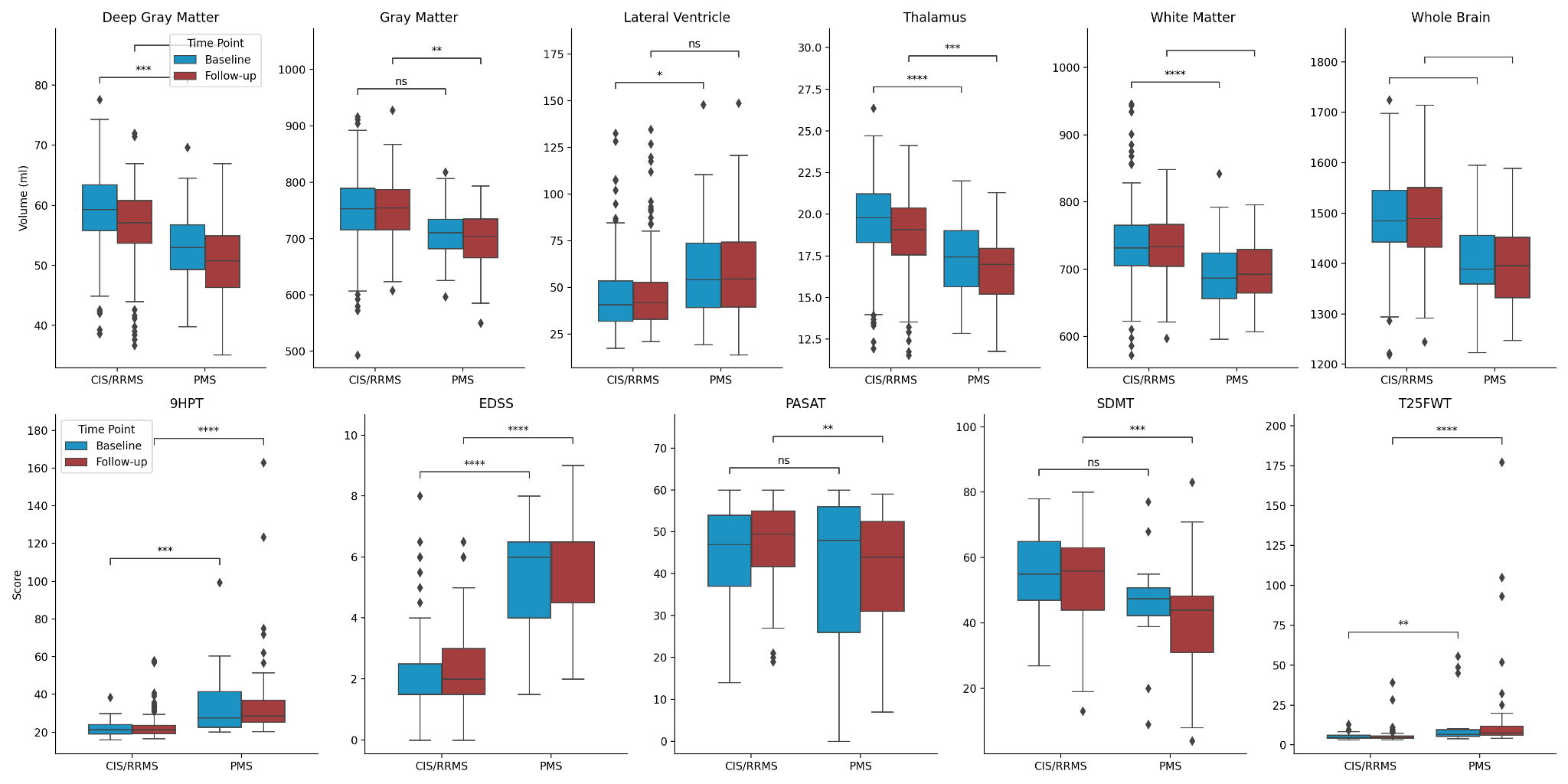


**Legend:** ANalysis of COVAriance (ANCOVA) was used to compare between the two subgroups. p-value annotation legend: ns: 5.00e-02 < p <= 1.00e+00, *: 1.00e-02 < p <= 5.00e-02, **: 1.00e-03 < p <= 1.00e-02, ***: 1.00e-04 < p <= 1.00e-03, ****: p <= 1.00e-04.

**Supplement Figure 4.** Changes in blood serum biomarker concentration between patients with Progressive MS (PMS) and Clinically Isolated Syndrome (CIS) and Relapsing-Remitting MS (RRMS).


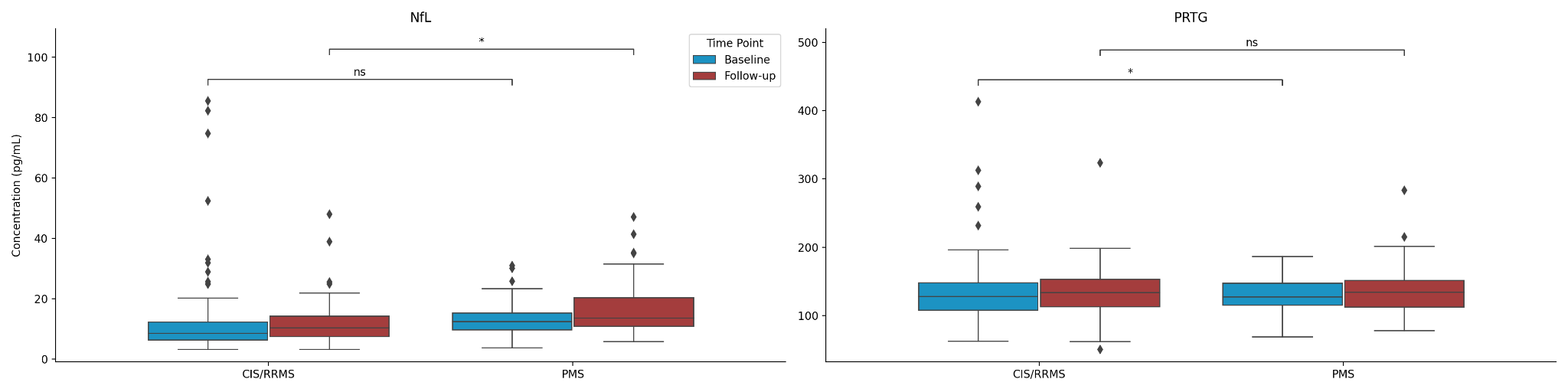


ANalysis of COVAriance (ANCOVA) was used to compare between the two subgroups. p-value annotation legend: ns: 5.00e-02 < p <= 1.00e+00, *: 1.00e-02 < p <= 5.00e-02, **: 1.00e-03 < p <= 1.00e-02, ***: 1.00e-04 < p <= 1.00e-03, ****: p <= 1.00e-04.
